# Systemic inflammation, enteropathogenic *E. Coli*, and micronutrient insufficiencies in the first trimester as possible predictors of preterm birth in rural Bangladesh: a prospective study

**DOI:** 10.1186/s12884-024-06266-9

**Published:** 2024-01-24

**Authors:** Meghan K. Gerety, Debora K. Kim, Rebecca M. Carpenter, Jennie Z. Ma, Christian Chisholm, Mami Taniuchi, Md Ohedul Islam, Suporn Pholwat, James A. Platts-Mills, Md Shahjahan Siraj, Sk Masum Billah, Rashidul Haque, William A. Petri

**Affiliations:** 1 Department of Biostatistics, Epidemiology & Informatics, Perelman School of Medicine, University of Pennsylvania, Philadelphia, PA USA; 2 Department of Obstetrics and Gynecology, University of Texas Medical Branch, Galveston, TX USA; 3 Department of Public Health Sciences, University of Virginia School of Medicine, Charlottesville, VA USA; 4 Department of Obstetrics and Gynecology, University of Virginia School of Medicine, Charlottesville, VA USA; 5 Division of Infectious Diseases and International Health, University of Virginia School of Medicine, Charlottesville, VA USA; 6 The International Centre for Diarrhoeal Disease and Research, Bangladesh (icddr,b), Dhaka, Bangladesh; 7 Sydney School of Public Health, The University of Sydney, Sydney, Australia

**Keywords:** Inflammation, anemia, Early pregnancy, Enteric pathogens, Premature birth

## Abstract

**Background:**

An incomplete understanding of preterm birth is especially concerning for low-middle income countries, where preterm birth has poorer prognoses. While systemic proinflammatory processes are a reportedly normal component of gestation, excessive inflammation has been demonstrated as a risk factor for preterm birth. There is minimal research on the impact of excessive maternal inflammation in the first trimester on the risk of preterm birth in low-middle income countries specifically.

**Methods:**

Pregnant women were enrolled at the rural Bangladesh site of the National Institute of Child Health Global Network Maternal Newborn Health Registry. Serum samples were collected to measure concentrations of the inflammatory markers C-reactive protein (CRP) and Alpha-1-acid glycoprotein (AGP), and stool samples were collected and analyzed for enteropathogens. We examined associations of maternal markers in the first-trimester with preterm birth using logistic regression models. CRP and AGP were primarily modeled with a composite inflammation predictor.

**Results:**

Out of 376 singleton births analyzed, 12.5% were preterm. First trimester inflammation was observed in 58.8% of all births, and was significantly associated with increased odds of preterm birth (adjusted odds ratio [aOR] = 2.23; 95% confidence interval [CI]: 1.03, 5.16), independent of anemia. Maternal vitamin B12 insufficiency (aOR = 3.33; 95% CI: 1.29, 8.21) and maternal anemia (aOR = 2.56; 95% CI: 1.26, 5.17) were also associated with higher odds of preterm birth. Atypical enteropathogenic *E. coli* detection showed a significant association with elevated AGP levels and was significantly associated with preterm birth (odds ratio [OR] = 2.36; 95% CI: 1.21, 4.57), but not associated with CRP.

**Conclusions:**

Inflammation, anemia, and vitamin B12 insufficiency in the first trimester were significantly associated with preterm birth in our cohort from rural Bangladesh. Inflammation and anemia were independent predictors of premature birth in this low-middle income setting where inflammation during gestation was widespread. Further research is needed to identify if infections such as enteropathogenic *E. coli* are a cause of inflammation in the first trimester, and if intervention for infection would decrease preterm birth.

## Background

Preterm birth (PTB), defined as live births delivered < 37 completed weeks of gestation per World Health Organization, is a leading cause of neonatal morbidity and mortality worldwide [1]. PTB is estimated to be experienced by around 15 million neonates globally and is disproportionately increased in South Asia, Africa, and North America [2]. The adverse pregnancy outcome is largely idiopathic, and previously reported multifactorial contributors of PTB such as infection, diet, anemia and others make preterm birth a global burden that is not yet clearly understood [3]. The consequences of an incomplete understanding of PTB are especially high for citizens of low-middle income countries (LMICs), for whom preterm birth tends to have poorer prognoses for neonatal mortality and quality of life. Therefore, it is imperative to elucidate determinants of preterm births to predict and identify patients at higher risk of PTB and for improved management during gestation.

Systemic proinflammatory processes have been reported to increase in the first trimester as a physiologically normal process of gestation, facilitating placental development and implantation [4–6]. However, excessive inflammation, which signifies an imbalance between fetal-maternal tolerance and inflammation hypothesized to facilitate implantation in gestation, has been reported as a risk factor for PTB [4, 7]. Specifically, a serum C-reactive protein level greater than or equal to 8 mg/l has been previously used to distinguish expected versus greater than expected systemic inflammation [8, 9]. Additionally, it has been hypothesized that elevated proinflammatory cytokines lead to increased prostaglandin production, thereby prompting preterm labor via premature cervical ripening and uterine contractions [10, 11]. While multiple studies have shown increased inflammation as associated with increased risk of PTB, the role of excessive maternal inflammation in the first trimester in elevating risk of PTB in LMICs specifically remains largely unclear [9, 12–14]. Data on preterm births is disproportionately available from high-income countries, and data that is available in LMICs is of variable quality and often sourced from urban areas that are not nationally representative [15]. To our knowledge, there have been limited studies reported on the significance of inflammation during the first trimester of gestation on adverse birth outcomes in a LMIC setting.

Markers of inflammation have been useful tools for the surveillance of pregnant women undergoing acute and chronic systemic inflammation. Maternal serum concentration of high-sensitivity C-reactive protein (CRP) has been used as an effective marker of inflammation resulting from acute and chronic infective and non-infective conditions [9, 16]. CRP is synthesized primarily in hepatocytes but also in macrophages, lymphocytes, and endothelial cells in response to proinflammatory cytokines, mainly IL-6, IL-1 and TNF-a [17]. Alpha-1-acid glycoprotein (AGP) is another acute phase reactant which has consistently been demonstrated to reflect chronic inflammation [18]. AGP rises at a slower rate, then stays elevated longer in serum compared to CRP [19]. When measured in conjunction, AGP and CRP can be useful in detecting subclinical inflammation [16]. However, few studies have considered both AGP and CRP in relation to preterm birth. In this paper, CRP and AGP levels were analyzed to elucidate the role of first trimester inflammation on preterm births in rural Bangladesh.

Insufficiencies in micronutrients such as vitamin A, B12 and folate have been previously identified to be associated with increased risk of PTB and other adverse pregnancy outcomes [20]. At large, however, studies have conflicting findings on whether micronutrient levels are indeed instrumental in predicting PTB, and if they do, the precise mechanisms by which they contribute to PTB are yet unclear [21]. Some studies have hypothesized that low vitamin A levels may cause individuals to be more susceptible to infection and, subsequently, to PTB [22, 23]. Furthermore, low vitamin B12 and folate have been suggested to impair placental function, predisposing mothers to PTB [21, 24–26]. Additionally, anemia specifically in the first trimester was shown in a meta-analysis to be associated with preterm birth [27]. Therefore, micronutrient and hemoglobin levels were explored to delineate their possible associations with PTB, independent of inflammation in this LMIC setting. Furthermore, enteric pathogens were explored to identify pathogens associated with increased systemic inflammation and with PTB. Our findings on associations between odds of preterm birth and systemic inflammation may aid risk stratification early in pregnancy, thereby assisting improved management of PTB in LMICs.

## Methods

### Data sources and sampling procedure

Subjects were enrolled from a sub-study of the National Institute of Child Health Global Network Maternal Newborn Health Registry (MNHR) (ClinicalTrials.gov Identifier: NCT01073475). The sub-study focused on the Bangladesh MNHR site, which was established at the rural Ghatail and Kalihati sub-districts of the Tangail district [28]. Objectives and procedures for the Bangladesh MNHR site have been described in detail previously [28]. Between October 6, 2021, and February 24, 2022, 1637 participants were enrolled in the registry. Of these participants, 463 who were enrolled during the first trimester (< 13 weeks gestational age) of pregnancy were randomly selected and asked to provide additional blood samples. Forty-six did not consent to blood sample collection, and one participant who did consent was later determined to have a false pregnancy and excluded. This resulted in 416 study participants who consented to the collection of additional blood samples; 370 of these participants consented to the collection of additional stool samples. Both samples were collected on the same day. Trained MNHR registry administrators collected sociodemographic and obstetric data including age, education level, household fuel type, household assets, parity, and last menstrual period through interview-administered questionnaires in the local language during a home visit upon registry enrollment. Height (m) and weight (kg) were measured at enrollment screenings. Birth date (used to calculate gestational age at delivery) and delivery mode were obtained through interview of the mother within 72 h of birth. Birth weight and baby sex were recorded by trained field workers within 72 h of birth. Evidence of maternal hypertensive disease was obtained from medical records from doctor’s visits or delivery.

### Laboratory analysis

Venous or capillary blood was collected from each participant during an antenatal care visit at the Kalihati Upazilla Health Complex, Kalihati, Bangladesh. Hemoglobin concentration was determined locally by a complete blood count (CBC) test performed with an automated cell counter (Mindrey BC_2800) and serum was separated and stored at -20 degrees Celsius at the International Centre for Diarrhoeal Disease Research, Bangladesh (icddr,b). Serum CRP, AGP, and retinol binding protein 4 (RBP4) concentrations were assessed by sandwich ELISA. Serum folate and vitamin B12 were analyzed by electrochemiluminescence immunoassay on a fully-automated Cobas e601 analyzer (Roche Diagnostics GmbH, Mannheim, Germany).

Stool total nucleic acid was extracted from 368 stool samples with the QIAamp Fast DNA stool mini kit (Qiagen, Hilden, Germany) following a bead beating step [29]. Phocine herpesvirus (PhHV) and bacteriophage MS2 served as extrinsic controls and were spiked to the lysis buffers to monitor extraction and amplification. Extraction blanks were used to monitor contamination during extraction and no-template controls were used to monitor contamination during PCR. Reverse transcriptase quantitative polymerase chain reaction (RT-qPCR) testing was performed with a custom TaqMan Array Card (TAC) (Thermo Fisher Scientific, Carlsblad, California) [30]. The card included all enteropathogen targets previously analyzed by TAC in the Global Enteric Multicenter Study (GEMS) [31].

### Preterm birth and maternal characteristic definitions

Gestational age (GA) was determined from the last menstrual period (LMP). LMP calendars were utilized to improve accuracy of LMP reporting and women at risk for pregnancy were surveilled by home visits every two months [28]. Preterm birth was defined as gestational age at delivery less than 37 weeks. In exploratory analyses, gestational age at delivery was also categorized as before 34 weeks, between 34 and 37 weeks, and at least 37 weeks, similar to the approach of Pitiphat et al. [9].

Maternal serum CRP concentration was dichotomized, with elevated CRP defined as greater than 8 mg/L [9, 32]. Serum AGP concentration was also dichotomized, with AGP greater than 1 g/L constituting elevated levels [33], as listed in Table 1. These two variables were dichotomized to facilitate easier comparison to published literature that routinely uses cutoffs to differentiate between high and low levels of inflammation [9, 33]. A binary inflammation predictor was created to capture those with at least one inflammatory biomarker (elevated CRP or elevated AGP). Other first-trimester biomarkers were dichotomized by established clinical cutoffs (enumerated in Table 2), including RBP4 concentration [33], vitamin B12 [34], CBC hemoglobin [35], and folate [36, 37]. Maternal age and parity at enrollment were grouped into categorical variables to capture clinically significant cut points, seen in Table 1. Maternal body mass index (BMI; kg/m^2^) was computed from height and weight measurements. A composite score using principal component analyses of household assets was used to construct a five-level wealth index variable. Household fuel was considered separately from wealth index as a measure of pollution exposure. Evidence of hypertensive disease or pre-eclampsia was extracted from the medical records of local prenatal care and delivery providers by follow-up field staff; specific criteria were not included in their reports.

**Table 1:** Maternal characteristics at first-trimester enrollment and birth outcomes for 376 singleton deliveries in Bangladesh

	Not Preterm ( $\ge$37 wks) *n* = 329	Preterm (< 37 wks) *n* = 47	*P*-value^a^
**Maternal Characteristics at Enrollment** ^**b**^			
CRP concentration > 8 mg/L	120 (36.5)	25 (53.2)	0.041*
AGP concentration > 1 g/L	121 (36.8)	26 (55.3)	0.023*
BMI (kg/m^2^)			0.117^+^
Mean (SD)	21.72 (3.55)	22.65 (5.13)	
Underweight (< 18.5)	64 (19.5)	12 (25.5)	
Healthy weight (18.5–24.9)	210 (63.8)	22 (46.8)	
Overweight (25.0-29.9)	48 (14.6)	8 (17.0)	
Obese ($\ge$30.0	7 ( 2.1)	5 (10.6)	
Age (years)			0.234
< 18	51 (15.5)	3 (6.4)	
18–35	269 (81.8)	43 (91.5)	
> 35	9 (2.7)	1 (2.1)	
Education (years)			0.102
No Education	11 (3.3)	4 (8.5)	
1–5	60 (18.2)	13 (27.7)	
6–10	191 (58.1)	24 (51.1)	
> 10	67 (20.4)	6 (12.8)	
Parity			0.013*
0	137 (41.6)	11 (23.4)	
1–2	164 (49.8)	27 (57.4)	
> 2	28 (8.5)	9 (19.1)	
Household Fuel			0.266
Electricity/LPG	35 (10.6)	2 (4.3)	
Dung/Wood/Charcoal/Straw/ Shrubs/Grass/Agricultural Crops	294 (89.4)	45 (95.7)	
Wealth Index^c^			0.174
Poorest	65 (19.8)	11 (23.4)	
Poorer	60 (18.2)	14 (29.8)	
Middle	64 (19.5)	10 (21.3)	
Richer	68 (20.7)	7 (14.9)	
Richest	72 (21.9)	5 (10.6)	
**Birth Outcomes and Pregnancy Characteristics** ^**b**^			
Gestational Age at Birth (weeks)			< 0.001*
Median (IQR)	40 (3.0)	35 (3.5)	
Birth Weight (g)			< 0.001*
Low Birth Weight (< 2500 g)	70 (21.3)	28 (59.6)	
Baby Sex			1.000
Male	171 (52.0)	25 (53.2)	
Female	158 (48.0)	22 (46.8)	
Evidence of Maternal Hypertensive Disease	3 (0.9)	1 (2.1)	1.000
Delivery Mode			1.000
C-Section	193 (58.7)	28 (59.6)	
Vaginal	136 (41.3)	19 (40.4)	

^a^Chi-squared test for categorical variables; two-sample t-test for continuous variables with means reported; Wilcoxon test for continuous variables with medians reported

^b^Data are n (%) unless otherwise specified

^c^Categorized by composite score based on principle component analyses of household assets

*P-value below significance level of 0.05

^+^*P*-value computed for continuous BMI

**Table 2:** Associations between first-trimester maternal markers and preterm birth

			Unadjusted Model^a^		Multivariable Model^b^	
**Dichotomized Biomarker**	**Not Preterm (** ***n*** ** = 329)** n (%)	**Preterm** **(** ***n*** ** = 47)** n (%)	**OR** ^**c**^ **(95% CI)**	***P***-value	**aOR** ^**d**^ **(95% CI)**	*P*-value
Inflammation^e^	185 (56.2)	36 (76.6)	2.55 (1.29, 5.41)	0.010 *	2.23 (1.03, 5.16)	0.049*
Anemia (Hemoglobin < 11 g/dL)	84 (25.5)	20 (42.6)	2.16 (1.14, 4.04)	0.016 *	2.56 (1.26, 5.17)	0.009*
Vitamin B12 Insufficiency (Vitamin B12 < 200 $\rho$mol/L)	24 (7.3)	10 (21.3)	3.43 (1.47, 7.58)	0.003 *	3.33 (1.29, 8.21)	0.010*
Vitamin A Insufficiency (RBP4 < 1.05 $\mu$mol/L)	64 (19.5)	5 (10.6)	0.49 (0.17, 1.19)	0.151	0.55 (0.17, 1.43)	0.258
Folate Deficiency (Folate < 6.7 nmol/L)	40 (12.2)	8 (17.0)	1.48 (0.61, 3.26)	0.353	1.35 (0.50, 3.34)	0.537

^a^Logistic regression models fit for each predictor individually without covariate adjustment

^b^Logistic regression model including all predictors listed in table, adjusted for baby sex (male or female), maternal BMI (kg/m^2^), maternal age (< 18 years, 18–35 years, or > 35 years), maternal years of education (> 10 years, 6–10 years, 1–5 years, or no education), maternal parity (> 2, 1–2, or 0), fuel types (dung/wood/charcoal/straw/shrubs/grass/agricultural crops, or LPG/electricity), delivery mode (C-section, or vaginal), maternal wealth index (poorest, poorer, middle, richer, richest), and evidence of hypertensive disease or pre-eclampsia (no or yes)

^c^Odds ratio

^d^Adjusted odds ratio

^e^Defined as elevated Alpha 1 AGP (serum concentration > 1 g/L) or elevated CRP (serum concentration > 8 mg/L)

*P-value below significance level of 0.05

### Statistical analysis

From the 416 pregnant women in the study, 386 births were recorded. This difference results from four twin deliveries in the cohort, plus miscarriages (*n* = 28), mothers dying before entering labor (*n* = 4), and medically terminated pregnancies (*n* = 2). Twin gestations were excluded from the analysis (*n* = 4), due to a baseline increased prevalence of PTB [38]. Mother-baby pairs were also excluded when no maternal folate measure was available (*n* = 2). Thus, the analytic dataset had a sample size of 376 mother-child dyads.

Maternal and delivery characteristics by preterm birth occurrence were compared through medians, means, and proportions. The distribution of CRP and AGP concentrations in mothers with deliveries before 34 weeks, between 34 and 37 weeks, and at least 37 weeks were compared with violin plots and pairwise Wilcoxon rank-sum tests, adjusted with the Holm-Bonferroni method [39].

Unadjusted and multivariable logistic regression models were fit for the binary preterm birth outcome, and odds ratios (ORs) and 95% confidence intervals (CIs) were estimated. The multivariable model included the composite inflammation predictor, anemia, vitamin B12 insufficiency, vitamin A insufficiency, and folate deficiency, and was adjusted for baby sex, maternal BMI, maternal age, maternal years of education, maternal parity, household fuel type, delivery mode, maternal wealth index, and evidence of hypertensive disease or pre-eclampsia. Further details follow Table 2. As a sensitivity analysis, a multivariable model with all aforementioned covariates except for those measured after the exposure assessment (delivery mode and evidence of hypertensive disease or pre-eclampsia) was performed. Interactions between anemia and inflammatory markers were considered and tested in multivariable models, as anemia can result from underlying inflammatory conditions [40].

Stool samples from 335 of the participants in the preterm birth analytic dataset were tested for enteric pathogens. Results for extrinsic controls and no-template controls were evaluated for quality control. Certain targets were collapsed; typical enteropathogenic *Escherichia coli* (tEPEC) was defined as strains positive for the EPEC *eae* gene and the *bpfA* gene targets but negative for both *stx1* and *stx2*, whereas atypical enteropathogenic *Escherichia coli* (aEPEC) was positive for the *eae* gene but negative for the *bfpA*, *stx1*, and *stx2* genes [41, 42]. The analysis focused on the most prevalent pathogens and (detected in $\ge$10% of mothers) in the interest of both statistical power and avoiding multiple comparisons. Enteric pathogens were analyzed by binary detection and by quantification cycle (Cq) values, which are an inverse metric for pathogenic load. Associations with elevated AGP and elevated CRP were evaluated through logistic regression models. Enteric pathogens found to be associated with either inflammatory marker were then tested for association with preterm birth via additional logistic regression models.

All statistical analyses were conducted in R (version 4.2.2).

## Results

### Participant characteristics

Out of 376 singleton births analyzed, 12.5% (*n* = 47) were preterm. The mean gestational age at birth for preterm deliveries was 33.49 weeks (SD = 3.71). Participant characteristics grouped by occurrence of PTB are shown in Table 1. The prevalence of elevated maternal CRP was higher for women who delivered preterm (55.3%) than for those who did not (36.5%). Elevated AGP was also more prevalent in women who delivered preterm (55.3%) versus those who did not (36.8%). Participants who delivered preterm were more likely to be less than 18 years old at enrollment, more likely to be nulliparous, and less likely to be considered “Richer” or “Richest”. The prevalence of other dichotomized biomarkers stratified by occurrence of preterm birth is included in Table 2. Mothers in this cohort who delivered preterm were more likely to have anemia, more than twice as likely to have vitamin B12 insufficiency, and less likely to have vitamin A insufficiency.

**Fig. 1:**
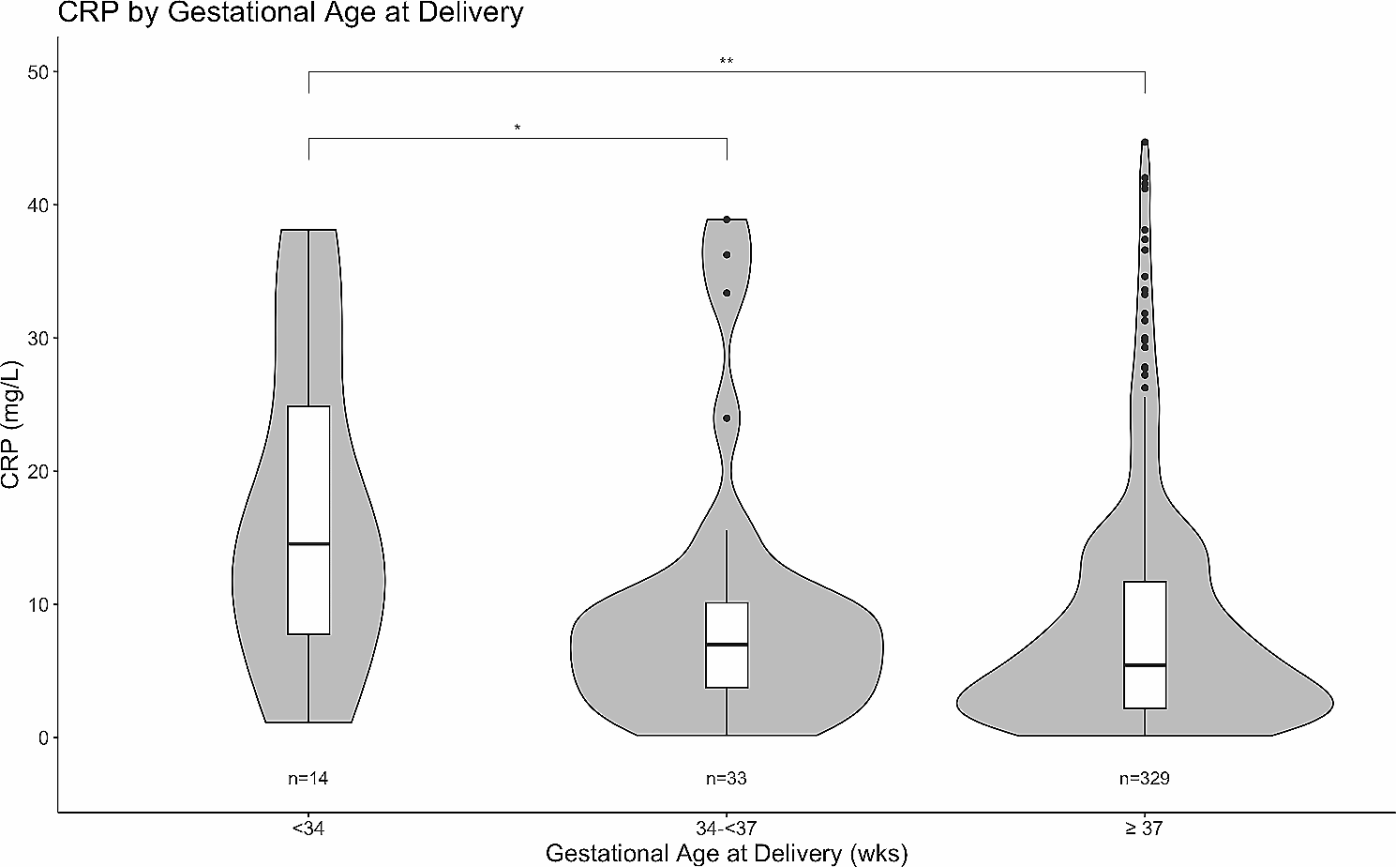
First-trimester maternal CRP levels grouped by gestational age at delivery. Distribution of first-trimester maternal CRP levels grouped by gestational age at delivery. Brackets indicate significant pairwise Wilcoxon rank-sum tests after Holm-Bonferroni adjustment: **adjusted *p*-value $\le$0.01, *adjusted *p*-value $\le$0.05

**Fig. 2:**
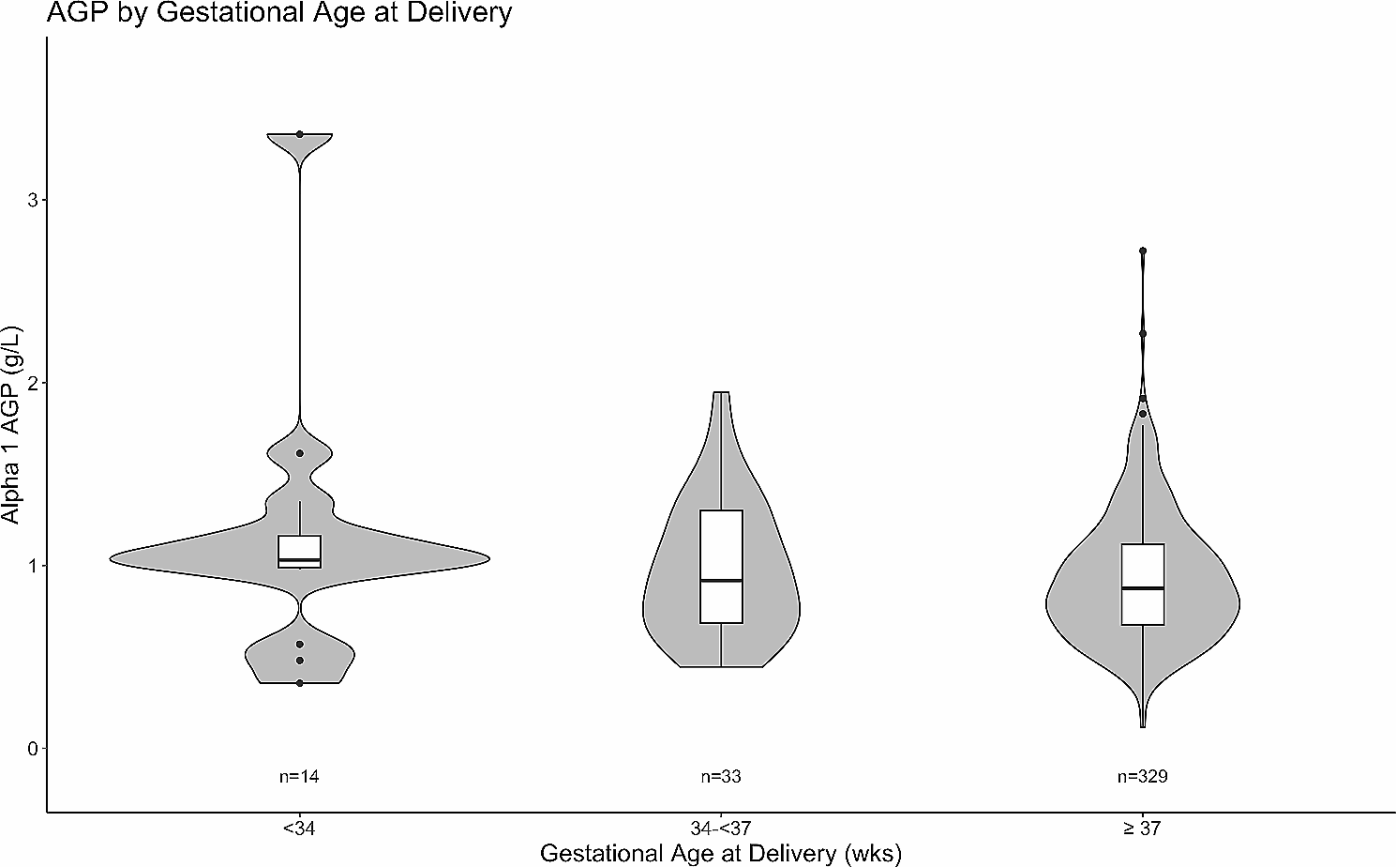
First-trimester maternal AGP levels grouped by gestational age at delivery. Distribution of first-trimester maternal AGP levels grouped by gestational age at delivery. No significant pairwise Wilcoxon rank-sum tests after Holm-Bonferroni adjustment.

Out of 47 observed preterm births, 29.8% (*n* = 14) occurred earlier than 34 weeks GA. Mothers who delivered before 34 weeks had significantly higher median maternal CRP in the first trimester than those who delivered between 34 and 37 weeks and those who delivered at or later than 37 weeks (Fig. 1). Median maternal AGP was similarly highest in the first trimester for women who delivered before 34 weeks GA, followed by those who delivered between 34 and 37 weeks GA, though differences were not statistically significant after adjustment (Fig. 2).

### Associations with preterm birth

Inflammation (elevated CRP or elevated AGP), anemia, and vitamin B12 insufficiency were significantly associated with increased odds of preterm birth in unadjusted logistic regression models (Table 2). Particularly, inflammation was significantly associated with a 123% increase in the odds of preterm birth (aOR = 2.23; 95% CI: 1.03, 5.16) (Table 2). In the same model, vitamin B12 insufficiency was significantly associated with a 223% increase in the odds of preterm birth (aOR = 3.33; 95% CI: 1.29, 8.21) and anemia was significantly associated with a 156% increase in the odds of preterm birth (aOR = 2.56; 95% CI: 1.26, 5.17). Neither folate deficiency nor vitamin A insufficiency showed significant associations with the odds of preterm birth in unadjusted or multivariable models. None of the odds ratios corresponding to covariates reached significance. The results of the sensitivity analysis that excluded delivery mode and evidence of hypertensive disease or pre-eclampsia were comparable. Inflammation (aOR = 2.22; 95% CI: 1.03, 5.13), anemia (aOR = 2.62; 95% CI: 1.30, 5.27), and vitamin B12 insufficiency (aOR = 3.23; 95% CI: 1.26, 7.91) were again significantly associated with preterm birth in this model. The interaction term between inflammation and anemia was insignificant when added to the full multivariable model (*p*-value = 0.97), and the adjusted odds ratios for inflammation stratified by anemia presence or absence were comparable (aOR = 2.20 when anemia was present, aOR = 2.26 when anemia was absent). The interaction term was thus excluded from the final adjusted model in Table 2.

### Maternal enteric pathogens

Overall, 77.2% of the 335 mothers had at least one enteric pathogen detected. Three of the collapsed targets considered were detected in more than 10% of analyzed mothers: aEPEC (28.4%), enteroaggregative *Escherichia coli* (EAEC; 27.2%), and Shiga toxin-producing *Escherichia coli* (STEC; 12.2%). Presence of aEPEC (OR = 1.70; 95% CI: 1.05, 2.76) and higher aEPEC load (OR = 0.94; 0.89, 0.98) were significantly associated with increased odds of elevated AGP (Table 3). Odds of elevated AGP were not associated with EAEC or STEC, and no significant associations with elevated CRP relative to these three bacteria were observed. Presence of aEPEC was significantly associated with increased odds of preterm birth (OR = 2.36; 95% CI: 1.21, 4.57), and higher loads of aEPEC were also significantly associated with increased odds of preterm birth (OR = 0.92 ;95% CI: 0.86, 0.98). When these models were adjusted for elevated AGP, the strength of association was slightly attenuated. Associations between the most prevalent enteropathogens and micronutrient deficiencies were considered, but did not reach statistical significance.

**Table 3:** Atypical EPEC associations with inflammatory markers and preterm birth

Characteristics or Model Result^a^	aEPEC Not Detected (*n* = 240)	aEPEC Detected (*n* = 95)	*P*-value	aEPEC Cq Value^b^	*P*-value
Elevated AGP (AGP concentration > 1 g/L)	90 (37.50)	48 (50.53)	-	-	-
OR (95% CI) for Elevated AGP	Ref	1.70 (1.05, 2.76)	0.030*	0.94 (0.89, 0.98)	0.012*
Elevated CRP (CRP concentration > 8 g/L)	94 (39.17)	37 (38.95)	-	-	-
OR (95% CI) for Elevated CRP	Ref	0.99 (0.61, 1.61)	0.970	1.00 (0.95, 1.05)	0.978
Preterm Birth	23 (9.58)	19 (20.00)	-	-	-
OR (95% CI) for Preterm Birth	Ref	2.36 (1.21, 4.57)	0.011*	0.92 (0.86, 0.98)	0.006*
aOR (95% CI) for Preterm Birth^c^	Ref	2.16 (1.10, 4.44)	0.024*	0.92 (0.87, 0.99)	0.018*

^a^Data are n(%) unless otherwise specified

^b^Lower Cq values indicate higher loads of aEPEC

^c^Adjusted for elevated AGP

*P-value below significance level of 0.05

## Discussion

In this study of singleton pregnancies in a LMIC setting, systemic inflammation in the first trimester was significantly associated with PTB. Importantly, presence of first trimester inflammation was observed in 76.6% of all preterm births and 56.2% of all term births. Research on first trimester inflammation and PTB outcomes in LMICs has been extremely limited likely due to the difficulty of surveillance during early pregnancy in these settings. However, our findings demonstrating significant associations between elevated CRP and AGP levels, specifically in the first trimester, and PTB are consistent with previous studies showing associations between PTB and inflammatory markers such as elevated CRP, IL-6, IL-8 and macrophage migration inhibitory factor (MIF) [9, 43–45]. Our findings do conflict with a United States-based study that did not detect an association between CRP levels specifically in the first trimester and risk of preterm birth [46]. This discrepancy may be due to the authors’ use of 10–18 weeks in gestational age to characterize early pregnancy CRP levels, whereas we used < 13 weeks for the same variable. Considering that the authors conclude decreasing levels of CRP throughout gestation as being associated with lower odds of PTB, their first trimester cohort may have had higher levels of CRP had their gestational age cut-off been set at < 13 weeks instead of 18 weeks. Additional considerations for the difference in findings are that Chen et al. had a smaller sample size of mothers delivering preterm compared to our study, a higher proportion of excluded mothers delivering preterm (due to data unavailability), and that our study was conducted in a high infection burden environment.

As inflammation was highly prevalent and significantly associated with PTB, we were interested in exploring potential pathogens responsible for the inflammatory response. We used RT-qPCR results from stool samples collected on the same day as the serum used to measure inflammation to evaluate enteropathogens that may explain the high levels of inflammation in the cohort, albeit minimally due to data limitations. Atypical EPEC showed a significant association with elevated AGP levels but was not associated with CRP. This is somewhat consistent with the conventional understanding that CRP is an acute-phase reactant which rises earlier during inflammation while AGP has demonstrated a more gradual increase representing a longer-term exposure to inflammatory processes [47]. It may be hypothesized that elevated AGP captures a history of enteropathogenic infection or colonization in very early pregnancy. Interestingly, Splíchalová et al. previously reported that when porcine amniotic epithelium was infected with EPEC in vivo, proinflammatory cytokine, TNF-a, was elevated and anti-inflammatory cytokine, IL-10, was attenuated in the amniotic fluid compared to the non-pathogenic *E. coli* infected group and the uninfected control [48]. *E. coli* has previously been linked to inflammatory states in the intestine, possibly because nitrate produced by a host’s inflammatory response can be utilized by *E. coli* [49, 50]. Consistent with our findings, atypical EPEC specifically was found to have a slightly stronger association with intestinal inflammation (as measured by stool myeloperoxidase) than typical EPEC in the multisite MAL-ED study [51]. The precise nature of the relationship between *E. coli* and inflammation is still emerging, but our finding of an association between aEPEC and elevated AGP in mothers, which may further link aEPEC to preterm birth, demonstrates the importance of further study on the association between inflammation and specific *E. coli* strains. Additionally, the role of other potential infections in the first trimester such as asymptomatic bacteriuria, urinary tract infections, sexually transmitted infections and tuberculosis, previously reported to be associated with PTB, may be further elucidated [52–56]. This research is particularly needed in high infection burden environments where the rates of adverse pregnancy outcomes continue to cause significant neonatal morbidity and mortality. Ultimately, a more robust understanding of infectious processes contributing to PTB will help develop strategies to mitigate adverse outcomes associated with infection during the first trimester.

Our finding that anemia in the first trimester is significantly associated with increased odds of PTB is consistent with a study from Southern India that showed infants born to anemic mothers had a twofold higher risk of PTB [57], as well as a systematic review and meta-analysis of 18 studies showing that maternal anemia in the first trimester increases the risk of premature birth [58]. Notably, all interaction terms between elevated inflammatory markers and anemia were insignificant. This suggests that anemia may be associated with PTB independent of inflammation. Conversely, inflammation is associated with PTB independent of anemia. These results highlight early inflammation and anemia as considerations of maternal health which must be individually emphasized during gestation as addressing one factor may not guarantee reduction of risks caused by the other. From the perspective of surveillance and management, the presence of either early inflammation or anemia may be sufficient to raise concern as both are significantly associated with PTB.

In addition, our analyses showed that vitamin B12 insufficiency was significantly associated with PTB. Concordantly, a linear association between low maternal B12 and risk of PTB, wherein one SD increase in B12 predicted a relative risk of 0.89 for PTB, was reported in a meta-analysis by Rogne et al. [21]. Vitamin B12 is a cofactor especially relevant in the context of pregnancy as vitamin B12-dependent methionine synthetase is critical for DNA synthesis and erythropoiesis [24]. Although the exact mechanism by which insufficient vitamin B12 stores increases risk of PTB remains unclear, elevated serum levels of total homocysteine subsequent to decreased availability of B12 has been associated with deleterious downstream effects in pregnancy possibly via endothelial cell injury and disrupted placental development [21, 24–26]. Here we have seen that even among other prominent nutrition deficiencies, a low B12 level during early pregnancy is a strong predictor of preterm birth in LMIC settings.

A few limitations of our study stem from a lack of available data in this rural low resource setting. The crosstalk between diabetes and inflammation is particularly pertinent in our study of inflammation’s association to PTB. Diabetes mellitus has been previously reported as a systemically proinflammatory state, and elevated CRP in the first trimester has been significantly associated with developing gestational diabetes [59, 60]. Pertaining to our study population, a 2020 study showed that the prevalence of diabetes mellitus is estimated at 30.8% in rural and 35.9% in urban Bangladesh [61]. Additionally, a gestational diabetes prevalence of 9.7% and an increasing proportion of women who are overweight during pregnancy have been reported previously [62, 63]. Therefore, studying the association of diabetes with inflammation and with PTB in this study was considered. However, fasting blood sugar levels were unobtainable in our cohort and hemoglobin A1c values were outside the scope of this study. Similarly, for the evidence of hypertensive disease or pre-eclampsia, misclassification is a concern. This data was extracted from medical records, but the specific criteria used in determining whether evidence of hypertension or pre-eclampsia was present were not available. Given the low prevalence of this variable in Table 1 (0.9% for term births and 2.1% for preterm births), we suspect that not all cases were identified under this condition. However, misclassification of this variable would not alter the main findings of this study. Lastly, the use of last menstrual period (LMP) to determine gestational age by nature has limitations such as inaccurate recall, delayed ovulation, and bleeding from causes other than menstruation. However, in this rural setting where ultrasound coverage is low, LMP serves as a more reliable tool for gestational age determination due to our ongoing, prospective, bi-monthly surveillance of married women conducted by trained field workers [28].

Additionally, the longitudinal analyses of EPEC throughout pregnancy would add valuable information regarding the nature of the association between this pathogen and preterm birth, however longitudinal stool samples were outside the scope of this study. Additionally, while our analyses may have been strengthened by analyzing or modeling births < 34 weeks gestation, there were not enough instances to do so reliably (*n* = 14). Nonetheless, this study does show a trend in Fig. 1 with CRP and AGP further elevated in mothers who deliver at < 34 weeks gestational age versus mothers who deliver moderate to late preterm (34–37 weeks). Modeling the relationships between inflammatory markers and size for gestational age was also of interest, and elevated CRP was associated with large for gestational age on univariate analysis. However, it was hypothesized that maternal diabetes was an important confounder in these relationships. Since fasting blood sugar levels were not recorded, maternal diabetes could not be adjusted for in the analysis. These relationships were not further examined due to this concern.

## Conclusion

In summary, this study of singleton pregnancies in a LMIC setting demonstrated that inflammation in the first trimester, reflected by elevated AGP or CRP, was significantly associated with preterm birth. To our knowledge, this is the first study to demonstrate a consistent finding of early gestational inflammation inferring an increased odds of PTB in a LMIC setting where inflammation during gestation is widespread. Further research is needed to identify associated bacteria such as atypical EPEC in women determined to be at higher risk of preterm birth due to elevated markers of inflammation in the first trimester and determine appropriate follow-up interventions. Earlier interventions for infections may decrease systemic inflammation and potentially affect the maternal and fetal immune environment with implications for PTB.

## Data Availability

All deidentified data will be made available to qualified investigators from the corresponding author upon reasonable request.

## Abbreviations

PTB: Preterm birth
CRP: C-reactive protein
AGP: Alpha-1-acid glycoprotein
LMIC: Low-middle income countries
MNHR: National Institute of Child Health Global Network Maternal Newborn Health Registry
icddr,b: International Centre for Diarrhoeal Disease Research, Bangladesh
LMP: Last menstrual period
GA: Gestational age
RT-qPCR: Reverse transcriptome quantitative polymerase chain reaction
OR: Odds ratio
aOR: Adjusted odds ratio
CI: Confidence Interval
EPEC: enteropathogenic *Escherichia coli*
aEPEC: atypical EPEC
tEPEC: typical EPEC
EAEC: enteroaggregative *Escherichia coli*
STEC: Shiga toxin-producing *Escherichia coli*
